# Trial-Type Dependent Frames of Reference for Value Comparison

**DOI:** 10.1371/journal.pcbi.1003225

**Published:** 2013-09-12

**Authors:** Laurence T. Hunt, Mark W. Woolrich, Matthew F. S. Rushworth, Timothy E. J. Behrens

**Affiliations:** 1 Wellcome Trust Centre for Neuroimaging, University College London, London, United Kingdom; 2 Centre for Functional MRI of the Brain (FMRIB), University of Oxford, Oxford, United Kingdom; 3 Oxford Centre for Human Brain Activity (OHBA), University of Oxford Department of Psychiatry, Oxford, United Kingdom; 4 Department of Experimental Psychology, University of Oxford, Oxford, United Kingdom; University College London, United Kingdom

## Abstract

A central question in cognitive neuroscience regards the means by which options are compared and decisions are resolved during value-guided choice. It is clear that several component processes are needed; these include identifying options, a value-based comparison, and implementation of actions to execute the decision. What is less clear is the temporal precedence and functional organisation of these component processes in the brain. Competing models of decision making have proposed that value comparison may occur in the space of alternative actions, or in the space of abstract goods. We hypothesized that the signals observed might in fact depend upon the framing of the decision. We recorded magnetoencephalographic data from humans performing value-guided choices in which two closely related trial types were interleaved. In the first trial type, each option was revealed separately, potentially causing subjects to estimate each action's value as it was revealed and perform comparison in action-space. In the second trial type, both options were presented simultaneously, potentially leading to comparison in abstract goods-space prior to commitment to a specific action. Distinct activity patterns (in distinct brain regions) on the two trial types demonstrated that the observed frame of reference used for decision making indeed differed, despite the information presented being formally identical, between the two trial types. This provides a potential reconciliation of conflicting accounts of value-guided choice.

## Author Summary

There are several competing theories of how the primate brain supports the ability to choose between different opportunities to obtain rewards – such as food, shelter, or more abstract goods (e.g. money). These theories suggest that the comparison of different options is either fundamentally dependent upon regions in prefrontal cortex (in which representations of abstract goods are often found), or upon motoric areas such as pre-motor and motor cortices (in which representations of specific actions are found). Evidence has been provided in support of both theories, derived largely from studies using different behavioural tasks. In this study, we show that a subtle manipulation in the behavioural task can have profound consequences for which brain regions appear to support value comparison. We recorded whole-brain magnetoencephalography data whilst subjects performed a decision task. Value comparison-related 13–30 Hz oscillations were found in ‘goods space’ in ventromedial prefrontal cortex in one trial type, but in ‘action space’ in pre-motor and primary motor cortices in another trial type - despite information presented being identical across trial types. This suggests both decision mechanisms are available in the brain, and that the brain adopts the most appropriate mechanism depending upon the current context.

## Introduction

Accounts of how the brain supports value-guided decision-making have been characterised as lying along a continuous spectrum [1]. At one end of the spectrum, it is argued that decisions are a serial process, in which stimuli are first perceived, then assigned values and fed to a subsequent decision stage where comparison takes place [2], [3]. Evidence in favour of such a view comes from comparing the relative prevalence and timing of pre- and post-decision variables encoded during economic choice [4], [5]. At the other end of the spectrum, decisions are framed as a parallel process, in which valuation, decision formation and action selection proceed simultaneously [6], [7]. Such a hypothesis is supported by the representation of potential responses in motor regions prior to decision termination [8]–[11], from probing the motor system behaviourally during the evolution of a decision [12], [13], and by comparing the relative timing of motor preparation responses in free- and forced-choice decisions [14].

The diversity of accounts is perhaps a symptom of value correlates being isolated in many different brain regions [15] – such as medial prefrontal [16]–[21], parietal [22]–[24], and motoric [25], [26] structures – and also of the diverse *frames of reference* in which these value correlates have been found. For example, one prominent serial model of decision making proposes that value comparison occurs in the frame of reference of abstract goods, prior to the representation of choice [27]. This would most likely occur in regions such as orbitofrontal and ventromedial prefrontal cortex, where goods-space value correlates have been isolated [5], [18], [28]. By contrast, a prominent parallel model suggests that comparison may take place in the frame of reference of actions needed to obtain a certain outcome [7]. This comparison may occur in structures such as motor and premotor cortex, in which value-related neural signals tied to specific actions can be found [8], [14], [24], [29].

It remains unclear whether decision processes occur serially or in parallel, and whether decision formation is principally resolved in action- or goods-space. It is possible that each account may be partially true, or that decisions are reached via a consensus between different systems [30]. One further reconciliation between the accounts might propose that the mechanism of decision formation might be *task-dependent* – that is, the frame of reference in which value-related signals are observed might depend upon both the framing of the decision and the way the data are analysed. Whilst both systems may still operate in parallel, the sensitivity to detect signals in a particular frame of reference might be strongly influenced by the task used – and so the differences between the tasks used across different studies might explain why goods-space signals are observed in some studies, and action-space signals in others.

To test this hypothesis, we designed a task in which subjects faced two different, interleaved types of trial. Each trial type comprised formally identical decisions, but had information presented in a subtly different fashion.

In the first type of trial (‘comparison’ trial, as reported in [5]), both options were presented simultaneously and subjects were free to respond at any time. Importantly, such a trial can be solved in several different ways. Decision formation could be carried out in the frame of reference of action values, tied to both left and right options, presumably in late motoric structures. Alternatively, it could occur in the frame of reference of abstract goods, presumably in frontal structures such as orbitofrontal or ventromedial prefrontal cortex (VMPFC) before undergoing a goods-to-action transformation. Although it is noteworthy that items are rarely encountered exactly simultaneously in nature, it is also true that such paradigms have been the norm in many studies of value guided choice [4], [14], [16]–[18], [28], [31], [32]. We hypothesised, based on signals observed in these tasks [4], [27], [28], that value-related signals might be observed in a goods frame of reference in this condition, and also that these goods value signals might be localised in structures such as VMPFC.

In the second type of trial (‘sequential’ trial), each option was presented sequentially, with a delay between the presentation of the first and second option, and a further delay before subjects executed their response. There were thus two differences between this trial type and the ‘comparison’ trial: first, its sequential nature, and second, the additional delays before a response was allowed. Again, such a trial could either be solved in an action-space or a goods-space frame of reference. However, both sequential presentation of options and the imposition of a delay prior to response have previously been used in tasks where action value signals have been recorded [8], [10], [26], [33]. Thus, we hypothesised that these two manipulations, although subtle, might push subjects towards a strategy of integrating information across probability and magnitude on each action as it is presented, and contribute towards the representation of a subjective value of making that action. This would suggest that the decision process could take place in the frame of reference of integrated action values [3], [17], [34], or that decision formation might occur coincidentally with the planning of the action necessary to execute the choice [7]. Either of these possibilities would lead to value signals in an action-space frame of reference as the decision was being made, and these signals might be predicted to occur in later, motoric structures.

## Results

### Subject choice behaviour is similar across comparison and sequential trials

**Figure 1:**
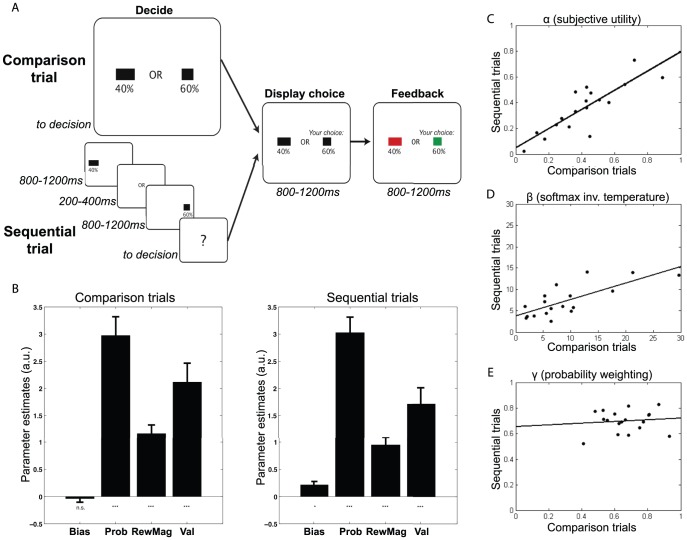
Experimental design and behavioural results. (A) Experimental timeline. The experiment contained two types of trial in which subjects chose between two risky prospects associated with differing reward magnitudes (bar widths) and reward probabilities (percentages). In ‘comparison’ trials, both options were presented simultaneously and subjects were free to respond as soon as they had made their decision. In ‘sequential’ trials, options were presented one after the other and subjects were free to respond once a question-mark appeared in the centre of the screen. (B) Logistic regression weights (mean +/− s.e.m.) of explanatory variables on choice behaviour on comparison trials (left) and sequential trials (right). (C) Prospect theory utility function parameters on comparison trials (ordinate) and sequential trials (abscissa); each datapoint represents the fit for an individual subject. Line shows least-squares fit to data (correlations reported in main text). (D) As (C), for softmax function parameters. (E) As (C), for probability weighting function parameters.

18 subjects completed 324 trials of each type, pseudorandomly interleaved, whilst undergoing magnetoencephalography (MEG). In ‘comparison’ trials, both options were presented simultaneously, until response. In ‘sequential’ trials, each option was presented sequentially, with a delay before a response was allowed (figure 1A). Subjects were not instructed to perform the task differently in each condition, except that in the sequential trials, they had to wait until the end of the delay period before they could respond (see Materials and Methods).

We first compared subject choice behaviour in comparison and sequential trials. We used logistic regression to test the influence of each option's reward probability and magnitude on subjects' choices during each type of trial (figure 1B). Reward probability, reward magnitude and their interaction each had a highly significant influence on subject choices (one-sample T-test on regression coefficients, all T(17)>4.6, all *p*<0.0005), but importantly there was no significant difference in these influences between the two types of trial (paired T-test on regression coefficients between trial types, all |T(17)|<1.52, all *p*>0.14). There was a slight bias towards choosing the second presented option on sequential trials (T(17) = 2.90, *p*<0.01), but no such bias towards choosing the left or right option on comparison trials (T(17) = −0.17, *p* = 0.87).

We also fit models from Prospect theory [35], [36] to describe subject choice behaviour on both types of trial (figure 1C–E). We fit a three-parameter model (α to describe curvature in subjective reward magnitude weighting, γ to describe non-linearities in subjective probability weighting, and β to describe stochasticity in choice behaviour) using maximum likelihood estimation. There was a strong correlation across subjects between α on sequential and comparison trials (figure 1A; *R* = 0.84,*p*<0.0001), and similarly for β (figure 1D; *R* = 0.75,*p*<0.0005), although no such correlation for γ (figure 1C; *R* = 0.11,*p* = 0.67). (This difference is potentially explained by the differing variances associated with the different parameters (coefficients of variation: α, 0.51; β, 0.70; γ, 0.17), which may imply that cross-subject variance in γ is primarily driven by noise in parameter fitting, rather than true variability in the population.) Importantly, there was no significant difference between fitted parameters on the two trial types, except for a trend towards α being larger in comparison trials (paired T-test, α: T(17) = 2.08, *p* = 0.052; β: T(17) = 1.58, *p* = 0.13; γ: T(17) = −0.956, *p* = 0.35).

In summary, behavioural results indicated that, even if subjects were to have adopted a different strategy in solving the two types of trial, their resultant choice behaviour was very similar in sequential and comparison trials.

### Transition from value representation to choice in motor cortex on ‘sequential’ trials

**Figure 2:**
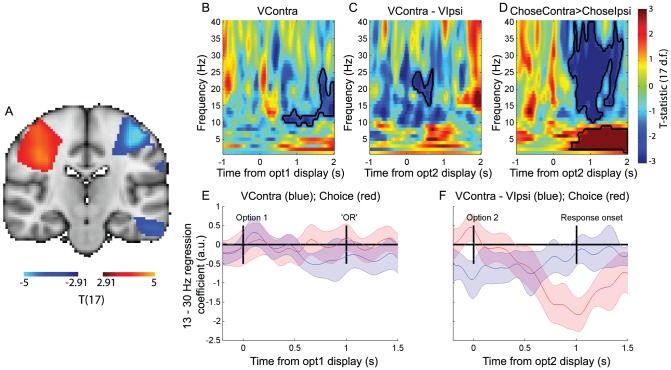
Motor cortex beta desynchronisation represents progression from value representation to choice on ‘sequential’ trials. (A) Statistical parametric map for contrast of beta band (13–30 Hz) activity for right buttonpresses>left buttonpresses, 500 ms–1000 ms after option 2 presentation (thresholded at T(17)>2.91, *p*<0.005 uncorrected, for display purposes). Warm colors reflect decreased beta desynchronisation in right hemisphere (ipsilateral to movement), cool colors reflect increased beta desynchronisation in left hemisphere (contralateral to movement). (B) Correlates of the value of option 1 at time of option 1 presentation, in hemisphere contralateral to option presentation. Color represents T-statistic; bordered areas reflect significant clusters (cluster-corrected *p*<0.05; permutation test). (C) Correlates of the value difference between the options contralateral and ipsilateral to the hemisphere, at the time of option 2 presentation. (D) Contrast of trials on which the chosen option is contralateral vs. ipsilateral to the hemisphere, at the time of option 2 presentation. (E) Timecourse of beta band correlates of value of contralateral option (blue and choice (red) at time of option 1 presentation. Lines represent mean +/− 95% confidence intervals across subjects. (Note that as 95% confidence intervals are plotted, rather than standard error of the mean (s.e.m.), error bars are ∼1.96 times wider than when plotting s.e.m.). (F) Timecourse of beta band correlates of value difference (blue) and choice (red) between options contralateral vs. ipsilateral to the hemisphere at time of option 2 presentation.

In both trial types, subjects chose left and right options with left and right thumbpresses respectively, allowing us to investigate decision formation in the frame of reference of actions by interrogating the timecourse of lateralised responses in motor cortices. We first investigated lateralised responses in sequential trials. We localised motor cortex by performing a contrast of right minus left planned responses, 500–1000 ms after the presentation of the second option (figure 2A). In the beta band (13–30 Hz), there was a greater degree of desynchronisation in the hemisphere contralateral to the planned movement (i.e. left hemisphere desynchonisation was greater on trials where a rightward movement was planned (peak T(17) = −5.59 (Montreal Neurological Institute (MNI) coordinates  = −36,−34,54 mm), T(17) = −5.86 (MNI = −50, −34, −54) whole-brain family-wise error corrected *p*<0.05)), and a lesser degree of desynchronisation in the hemisphere ipsilateral to the movement (i.e. right hemisphere desynchronisation was lesser on trials where a rightward movement was planned (peak T(17) = 4.63 (MNI = 56,0,34 mm)). This pattern of pre-movement beta desynchronisation is as would be expected from many previous studies of response selection [37]–[40].

Having localised this beta desynchronisation during movement preparation, we then investigated the temporal evolution of value correlates in the same region. In all analyses, we included the eventual categorical choice as a coregressor, to test whether signals were better predicted by value or by choice. At the time of option 1 presentation, beta desynchronisation (in the hemisphere contralateral to the side option 1 was presented on) was found to correlate with the value of this option; the higher the value, the more negative the beta power (figure 2B). As shown in figure 2B/E, this signal first emerged approximately 500 ms after stimulus presentation (significant cluster delineated by black line in figure 2B, tested via a cluster-based permutation test that corrects for multiple comparisons across time and frequency (see Materials and Methods)). It remained in the region throughout the delay period (in which the option was removed and replaced with a central word ‘OR’) (peak T(17) = −3.83, t = 1975 ms post-stimulus presentation, 14 Hz; cluster-corrected *p*<0.05, permutation test). The *negative* coefficient of the value correlate (shown in figures 2B and 2E) reflects *increased desynchronisation* [26], [37]–[40] in the beta band at the time of option 1. At the time of option 2 presentation, there was a negative correlate of the difference in value between the option contralateral to the hemisphere and the option ipsilateral to the hemisphere (figure 2C), with a significant cluster centred around 400 ms post-stimulus presentation; the greater the value difference between contralateral and ipsilateral options, the more negative the beta power (peak T(17) = −3.91, t = 325 ms post-stimulus, 23 Hz; cluster-corrected *p*<0.05). Such a signal is a value difference signal, but importantly it is tied to the frame of reference of a specific action (contralateral vs. ipsilateral movement), rather than the frame of reference of which option will be chosen on the current trial. (It is notable that part of this signal may be driven by the value of option 1, which is known prior to option 2 presentation – and so the effect of value difference may arise much earlier than when analysed time-locked to option 1 presentation. Indeed, when split into the separate subcomponents, option 1 influenced beta desynchronisation earlier than option 2 (see figure S1)).

**Figure 3:**
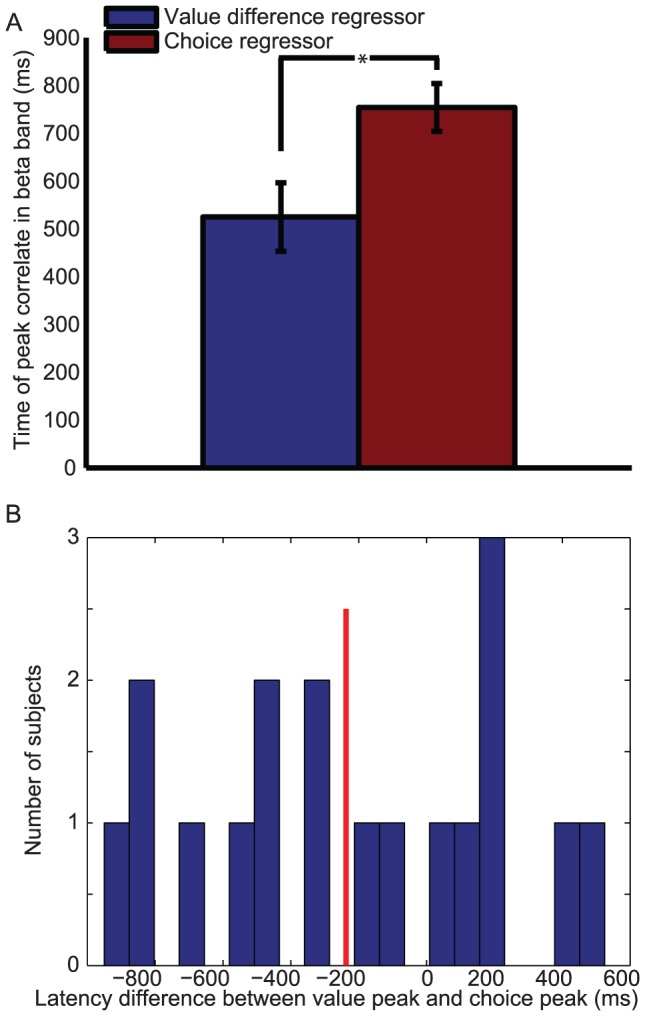
Relative latency of ‘action value difference’ and ‘choice’ effects (both in motor cortex beta desynchronisation) after stimulus 2 presentation on ‘sequential’ trials. (A) Comparison of the latency of the peak correlate of ‘value difference’ regressor in motor cortex beta desynchronisation (blue) against the latency of the peak correlate of the ‘categorical choice’ regressor in motor cortex beta desynchronisation (red). * denotes *p*<0.05, paired T-test across 18 subjects. (B) Histogram of individual subjects' latency differences between ‘value difference’ peak latency and ‘categorical choice’ peak latency; red line denotes median latency across subjects.

Using the regressor for categorical choice, we also identified a signal reflecting the categorical commitment to a rightward or leftward action (figure 2D) in the same region of interest, with beta desynchronisation being more negative when choices (button presses) were made to the side contralateral to the hemisphere than to the side ipsilateral to the hemisphere. Such a finding is unsurprising, as the region of interest was selected on the basis of differential beta desycnchronisation on left vs. right buttonpresses. However, the critical test is the *timing* of this categorical decision signal (figure 2D) *relative* to the action value signals (figure 2B/C). When the first option was presented (figure 2E), beta descynchronisation was explained by the value of the contralateral option (blue line) over and above any possible variance that could be attributed to the eventual choice that the subject would make (red line). This was because both value and choice regressors were included in the same multiple regression model, and whereas value correlates were significantly different from zero, choice correlates were not. From figure 2F, we see that in a similar multiple regression model, the categorical decision signal emerged prior to the time at which subjects were allowed to make their response, but after the value difference signal (peak T(17) = −7.36, t = 775 ms post-stimulus, 18 Hz). This suggests a transition from initially representing action value difference, to subsequently representing categorical choice. We formally compared the relative timing of these two signals by comparing the time of the peak T-statistic in each subject for the two signals (figure 3); this confirmed that the value-related signal preceded the categorical decision signal (paired T(17) = 2.14, *p*<0.05).

The relative timing of these value-related and categorical choice signals may reflect two possibilities. It may suggest that in sequential trials, late motoric structures directly support the comparison of values tied to specific actions. Alternatively, it may be that an evolving decision process taking place in other cortical structures is continually biasing action preparation or planning in motor cortex. In either case, it is clear that value correlates are present in motor cortex before a categorical decision has been reached.

### Categorical representation of choice, but not value, in motor cortex on ‘comparison’ trials

**Figure 4:**
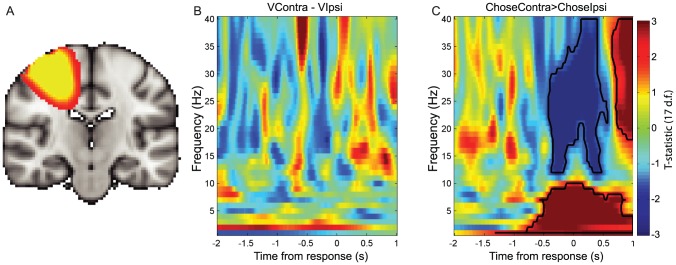
Motor cortex beta band desynchronisation reflects choice, but not value, on ‘comparison’ trials. (A) Statistical parametric map for contrast of beta band (13–30 Hz) activity for right buttonpresses>left buttonpresses, 500 ms–1000 ms after decision presentation (thresholded at T(17)>2.91, *p*<0.005 uncorrected, for display purposes). Warm colors reflect decreased beta desynchronisation in right hemisphere (ipsilateral to movement). (B) Correlates of value difference between the options contralateral and ipsilateral to the hemisphere, timelocked to the response. Color represents T-statistic; the absence of any bordered region reflects the absence of any significant clusters surviving multiple comparisons correction. (C) Contrast of trials on which chosen option was contralateral vs. ipsilateral to the hemisphere. Bordered areas reflect significant clusters (cluster-corrected P<0.05; permutation test).

We next investigated whether similar value signals could be seen prior to the representation of choice in comparison trials. Again, we found that 500–1000 ms after the decision was presented, there was a differential response for right versus left buttonpresses, with less beta band desynchronisation in the right hemisphere for rightward than for leftward movements (figure 4A; peak T(17) = 11.42, MNI = 28,−14,54, voxelwise whole brain corrected *p*<1*10^−5^). When searching for a correlate of the value of the options contralateral versus ipsilateral to the hemisphere, we timelocked to the response rather than the stimulus, as in this condition responses occurred at varying latencies rather than a fixed delay – and so, because reaction times correlate negatively with value difference [5], beta desynchronisation that was in fact associated with responses made at different latencies would give rise to spurious correlations with value.

Using this analysis, we found that there was no correlate of the difference in value between the option contralateral and the option ipsilateral to the hemisphere in the beta band, nor indeed in any frequency band from 1–40 Hz (figure 4B). By contrast, consistent with figure 4A, there was still a strong correlate of the categorical choice, with beta desynchronisation being more negative when choices were made to the contralateral side than to the ipsilateral side (figure 4C), peaking near the time of the response (peak T(17) = −5.46, t = 175 ms post-response, 26 Hz). Thus, on comparison trials, in contrast to the sequential trials, there was a categorical representation of choice but no lateralised representation of action value prior to the formation of the decision.

**Figure 5:**
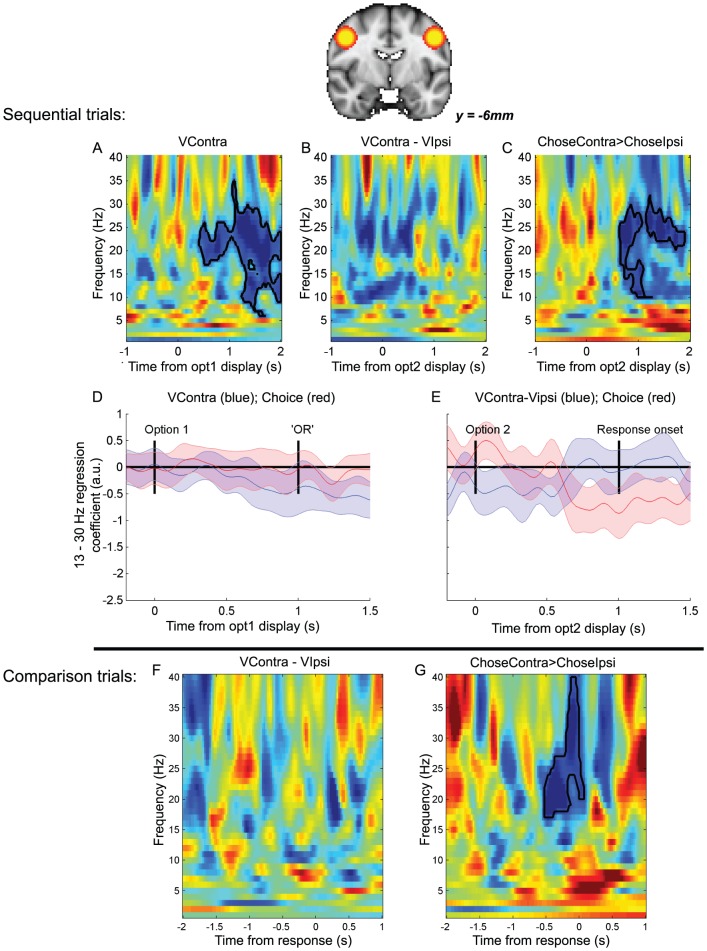
Lateral premotor cortex, similar to primary motor cortex, shows ‘action-space’ value followed by choice signals during sequential trials (A–E), and choice signal but no value signal during comparison trials (F–G). *Parts A–E are equivalent to parts B–F of* *figure 2*. (A) Correlates of the value of option 1 at time of option 1 presentation, in hemisphere contralateral to option presentation. Color represents T-statistic; bordered areas reflect significant clusters (cluster-corrected P<0.05; permutation test). (B) Correlates of the value difference between the options contralateral and ipsilateral to the hemisphere, at the time of option 2 presentation. (C) Contrast of trials on which the chosen option is contralateral vs. ipsilateral to the hemisphere, at the time of option 2 presentation. (D) Timecourse of beta band correlates of value of contralateral option (blue and choice (red) at time of option 1 presentation. Lines represent mean +/− 95% confidence intervals across subjects. (Note that as 95% confidence intervals are plotted, rather than standard error of the mean (s.e.m.), error bars are ∼1.96 times wider than when plotting s.e.m.). (E) Timecourse of beta band correlates of value difference (blue) and choice (red) between options contralateral vs. ipsilateral to the hemisphere at time of option 2 presentation. *Parts F–G are equivalent to parts B–C of* *figure 4*. (F) Correlates of value difference between the options contralateral and ipsilateral to the hemisphere, timelocked to the response. (G) Contrast of trials on which chosen option was contralateral vs. ipsilateral to the hemisphere.

On both sequential and comparison trials, we also found a similar set of signals emerged if we examined activity in lateral pre-motor, rather than primary motor, cortex (figure 5).

The absence of an action-value signal in comparison trials is a negative result, and so might be interpreted as a consequence of insufficient statistical power. To demonstrate that this was not the case, we used a formal interaction test (described below) and found a significant difference in action-value signals between the two conditions.

### Representation of value difference in ventromedial prefrontal cortex on ‘comparison’ trials

We then searched for response-locked correlates of value on comparison trials in ventromedial prefrontal cortex (VMPFC), a region we have previously identified as playing an important role in value comparison on these trials [5], [16]. We analysed data from the same region of VMPFC identified in our previous study [5], in which we found that (stimulus-locked) there was a temporal evolution from a representation of overall value to value difference in low frequencies (2–10 Hz). The location of this region of interest (MNI = 6,28,−6 mm) also lies within a cluster of activations identified in a recent meta-analysis of human functional MRI studies of value-guided choice [19]. We hypothesised, based on signals observed in other studies of this region, that it would not encode value in the frame of reference of actions, but of choice [5], [16], [18], [32] – which might be the result of a comparison occurring in ‘goods space’ [27], [28]. Based on our previous work [5], we also hypothesised that this region might particularly encode value on *‘harder’* trials, in which probability and magnitude advocate opposing choices, but not on *‘nobrainer’* trials, in which both probability and magnitude were both larger on the same side than on the other. Critically, we note that these harder trials are precisely those on which a comparison of attribute differences might be necessary to resolve the decision.

**Figure 6:**
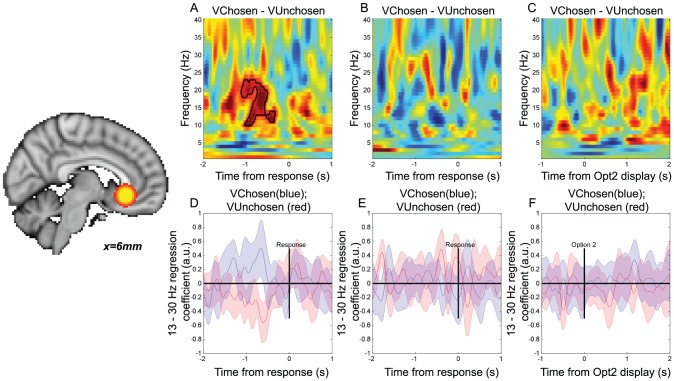
Ventromedial prefrontal cortex (VMPFC) beta band synchronisation reflects value difference on harder ‘comparison’ trials, but not on ‘sequential’ trials. (A) Correlates of the value difference between chosen and unchosen options, timelocked to the response, on harder comparison trials. Color represents T-statistic; bordered areas reflect significant clusters (cluster-corrected P<0.05; permutation test). (B) As (A), but for ‘nobrainer’ trials in which probability and magnitude advocated the same response. (C) Correlates of the value difference between chosen and unchosen options, timelocked to option 2 presentation, on harder sequential trials. (D) Separating the VMPFC beta band response on harder comparison trials reveals a positive correlate of the value of the chosen option (blue) and a negative correlate of the value of the unchosen option (red) prior to the response. Bars represent mean +/− 95% confidence intervals across subjects. (E) As (D), but for ‘nobrainer’ trials. (F) Separating the beta band response on harder sequential trials reveals no correlate of either chosen or unchosen value in VMPFC at the time of option 2 presentation.

**Figure 7:**
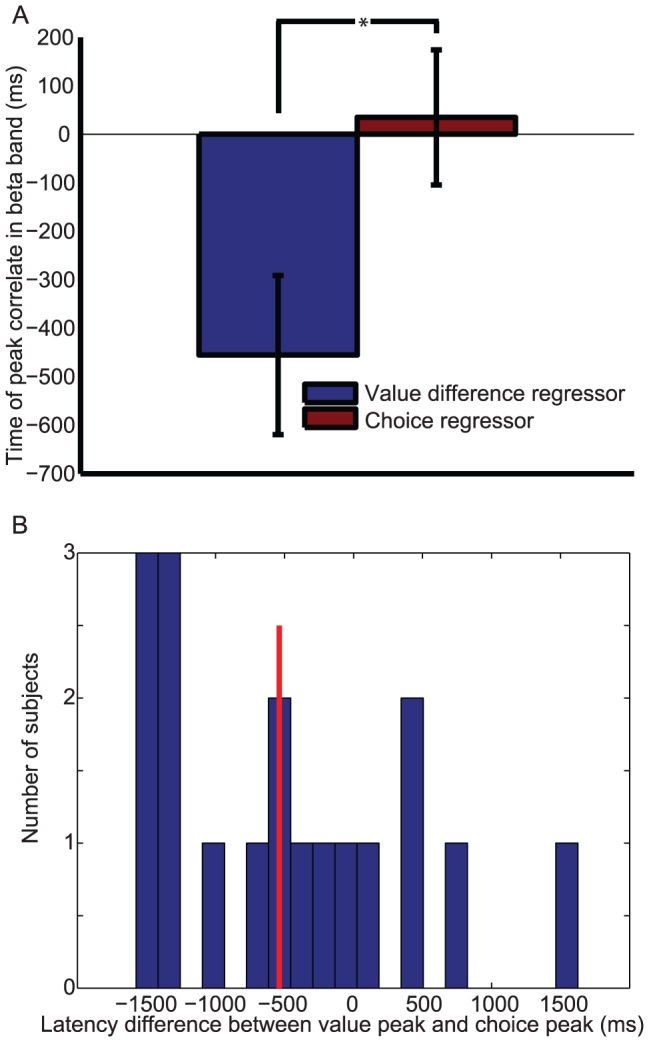
Relative latency of ‘goods value difference’ effect (in VMPFC beta synchronisation) and ‘choice’ effect (in motor cortex beta desynchronisation), timelocked to response on ‘comparison’ trials. (A) Comparison of the latency of the peak correlate of ‘value difference’ regressor in VMPFC beta synchronisation (blue) against the latency of the peak correlate of the ‘categorical choice’ regressor in motor cortex beta desynchronisation (red). * denotes *p*<0.05, paired T-test across 18 subjects. (B) Histogram of individual subjects' latency differences between ‘value difference’ peak latency and ‘categorical choice’ peak latency; red line denotes median latency across subjects.

On harder comparison trials, there was a positive correlate of the difference in value between chosen and unchosen options in the beta band approximately 750 ms prior to the response (figure 6A; peak T(17) = 4.05; t = 975 ms pre-response; F = 10 Hz). This value difference signal is in a different frame of reference to that isolated in primary motor cortex: it is not tied to the frame of reference of one or other specific action, but instead to the choice that is to be made. Importantly, when split into its subcomponents, this ‘goods-value’ signal contained both a positive correlate of the value of the chosen option and a negative correlate of the value of the unchosen option (figure 6D). Again, we formally compared the relative timing of this value-related signal in VMPFC to that of the categorical choice signal in motor cortex, by extracting the peak T-statistic for each signal in each subject (figure 7); we found that the VMPFC value related signal preceded the categorical motor signal (paired T(17) = 2.25, *p*<0.05).

**Figure 8:**
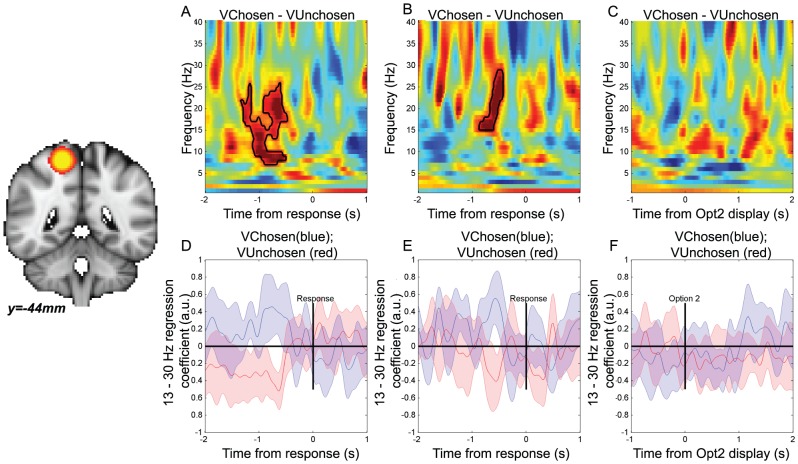
Right posterior superior parietal lobule, identified in our previous study of reward-guided decision making [5], shows beta correlates of chosen-unchosen value on both ‘harder’ and ‘nobrainer’ comparison trials, but not on harder sequential trials. *Parts A–F are equivalent to parts A–F of* *figure 6*. (A) Correlates of the value difference between chosen and unchosen options, timelocked to the response, on harder comparison trials. Color represents T-statistic; bordered areas reflect significant clusters (cluster-corrected P<0.05; permutation test). (B) As (A), but for ‘nobrainer’ trials in which probability and magnitude advocated the same response. (C) Correlates of the value difference between chosen and unchosen options, timelocked to option 2 presentation, on harder sequential trials. (D) Separating the pSPL beta band response on harder comparison trials reveals a positive correlate of the value of the chosen option (blue) and a negative correlate of the value of the unchosen option (red) prior to the response. Bars represent mean +/− 95% confidence intervals across subjects. (E) As (D), but for ‘nobrainer’ trials. (F) Separating the beta band response on harder sequential trials reveals no correlate of either chosen or unchosen value in pSPL at the time of option 2 presentation.

In contrast, on ‘nobrainer’ comparison trials, we found no significant correlation in VMPFC with the value difference between the chosen option minus the value of the unchosen option, nor of the subcomponents of this signal (figure 6B/E). However, this finding was complemented by signal in the posterior superior parietal lobule (pSPL), a region isolated in our previous study as showing similar dynamics to VMPFC in lower frequency bands (2–10 Hz), but across *both* harder *and* nobrainer trials [5]. In the beta band (13–30 Hz), pSPL showed a synchronisation that correlated positively with chosen-unchosen value across both harder (figure 8A/D) and nobrainer (figure 8B/E) trials, consistent with our previous study.

Finally, both VMPFC and pSPL also showed no correlation of chosen-unchosen value (or the separated subcomponents) on harder sequential trials (figure 6C/F; figure 8C/F), or nobrainer sequential trials. This finding is particularly important, as it suggests that when values are represented in ‘action space’ as a choice is being made (as was the case in sequential trials), there was no longer a detectable ‘goods space’ comparison in these regions. As before, we note that this is a negative result, and so we test it by comparing the strength of goods-value signals in each trial type formally below. We were also unable to detect any action value signal or categorical choice signals (equivalent to those observed in motoric structures above) in VMPFC or pSPL (figures S2 and S3).

### Formal contrast of effects in motor cortex and VMPFC in comparison and sequential trials

Finally, we formally compared the effect of value in VMPFC and motor cortex across the two trial types. In each subject, we extracted the peak T-statistic for the effect of value difference between the options in the ipsilateral and contralateral hemispheres from motor cortex, and the effect of value difference between chosen and unchosen options in VMPFC. We restricted our analysis to the beta band (13–30 Hz), focussing on the period from 2^nd^ stimulus onset up to 1 s post-stimulus on sequential trials, and from 1 s pre-response until response time on comparison trials. As the same frequency range was examined in each region/condition, and the regions of interest were isolated via orthogonal contrasts, this analysis was protected against circular inference [41]. A repeated measures ANOVA with independent variables of brain region (VMPFC/motor cortex) and trial type (comparison/sequential) revealed a significant interaction between these two variables on the peak effect of value (F_1,17_ = 7.29, *p*<0.02). *Post-hoc* T-tests revealed that there was a significantly greater effect of chosen-unchosen value in VMPFC on comparison trials than on sequential trials (paired T(17) = 2.42, *p*<0.05), and a slightly greater effect of ipsilateral-contralateral value in motor cortex on sequential trials than on comparison trials (paired T(17) = 1.83, *p*<0.05 one-tailed).

## Discussion

Conflicting accounts of value-guided choice have proposed that decision formation is either supported principally by comparing the value of alternative goods, or by the comparing the value of alternative actions. In the present study, we isolated evidence in support of both accounts, but in two distinct types of trial – one (comparison trials) in which goods-space value comparison signals were more readily apparent, and another (sequential trials) in which action value-space signals were found. These findings therefore present a possible reconciliation of the two accounts – that the brain adaptively adopts the strategy most appropriate to the current context.

The hypothesis that different tasks may be solved in different frames of reference may help to resolve apparently discrepant findings from previous studies in the literature. In one set of studies examining single unit activity during an economic choice task, Padoa-Schioppa and colleagues have identified dissociations between activity in orbitofrontal cortex (OFC) and anterior cingulate cortex (ACC). In this task, OFC neurons encode both pre- and post-decision variables, but not in the frame of reference of actions [28]. By contrast, ACC neurons encode solely post-decision variables, and are modulated by movement direction [4]. This has led to the hypothesis that in this task, items (here quantities of fruit juice) are compared in an abstract ‘goods space’ in OFC/VMPFC, before undergoing a goods-to-action transformation in ACC in order to implement the required action to obtain that item [27]. This hypothesis gains support from the presence of post-decision (chosen value) signals in VMPFC in a task in which a stimulus value-based comparison is made, but the action needed to implement the decision is not yet known [18]. On the other hand, it appears that when subjects are presented with tasks that can only depend upon learnt action values rather than stimulus values, then the structure critical for value-guided choice may change, with lesions to ACC and not OFC affecting behaviour [31], [42]. In even simpler forced-choice trials, on a task that does not require integration of information across multiple dimensions, there appears to be a temporal evolution from the initial coding of option values to the subsequent coding of action-related signals within relatively late, motoric structures, such as the supplementary eye fields [43]. In experiments where multiple possible actions are presented and held in working memory prior to a decision cue, enhanced representations of these actions can be seen prior to the decision in premotor cortex [8]. Similarly, when a free choice is made between alternative arm movements, evidence for a competitive decision mechanism (in the frame of reference of actions) is found in the parietal reach region [44]. Thus, in tasks where decisions in stimulus space or goods space are favoured, then neural correlates of the decision process is found in a stimulus- or goods-related frame of reference, whereas in tasks more closely tied to the comparison of different actions, correlates of the decision process appear in an action frame of reference. This observation unifies apparently discrepant findings as to the precise locus of decision-making processes in the brain.

On the other hand, the differing signals across the two trial types may not be a reflection of different neural mechanisms of choice being used in each context, but instead differential *sensitivity* to one or other mechanism in our analysis. For instance, it is possible that action value signals are present in motor cortices in all trials, but without a delay period they become too transient to be detected. Similarly, it is possible that the relatively weak sensitivity of MEG to deep anterior structures such as VMPFC [45] means that on sequential trials, any value comparison process that takes place over a space of several seconds is too weak to be detected. Future studies may address these questions by direct invasive recording from these structures, across different conditions.

Our findings from the sequential trials suggested one of two possibilities. One interpretation is that these trials were solved using a comparison of action values, as demonstrated by the transformation from a lateralised action value signal into a categorical choice signal in motor cortex beta band oscillations. An alternative account is that this signal is better interpreted as a (graded) motor planning signal, but there was a continual updating of this plan as a consequence of value comparison taking place elsewhere. Here, it is perhaps telling that many signals that have been interpreted as the intention to move by one set of researchers [46] have been related to decision-related signals by others [22].

In either case, these results have additional implications for our understanding of the role of motor cortex beta band oscillations in action selection. Whereas early accounts of these oscillations suggested that they might reflect an ‘idling rhythm’ [47], more recent suggestions have proposed that beta desynchronisations may reflect a change in the current sensorimotor set or *status quo* [38], or an increased likelihood of generating a novel voluntary action [39], [40]. Such proposals align with a role for decreases in beta band activity during response preparation, an idea corroborated by recent findings that lateralised beta reflects the accumulation of evidence for a leftward or rightward response during perceptual discrimination [9]. By contrast, a recent study has highlighted that lateralised beta band desynchronisation reflects the evidence for a particular response, rather than response preparation *per se*, whilst integrating evidence to make a decision [26]. The current findings on ‘sequential’ trials suggest a similar role for beta desynchronisation, as evidenced by the correlation with action value above and beyond any correlation with the categorical response that is going to be effected on a given trial (figure 2).

On comparison trials, we found that in the beta band, value comparison signals emerged in VMPFC (figure 6) that preceded categorical choice signals in primary motor cortex. Critically, we found that such signals were present on trials in which magnitude and probability advocated opposing choices (‘harder’ trials), but not on trials in which they both advocated the same choice (‘nobrainer’ trials). Such trials are those on which conflict between the two attributes comes into play, and attention must be guided to the attribute that is most salient for determining the current decision. Notably, this was not the case in the posterior superior parietal lobule (figure 8), in which goods value difference signals were present on both ‘harder’ *and* ‘nobrainer’ trials. This replicates findings in lower frequency ranges (2–10 Hz) from the same dataset [5], and may reflect an important difference between VMPFC and parietal cortex when considering value-guided choices with multiple attributes.

One further noteworthy difference between the signals observed in comparison and sequential trials is the relative timing of value difference and categorical choice signals in the two trial types. In sequential trials (figure 3), there was a median latency difference of approximately 200 ms between the peak of (action) value difference signal in motor cortex, and the peak of the categorical choice signal in the same region. By contrast, in comparison trials (figure 7), there was a median latency difference of around 500 ms between the peak of the (goods) value difference signal in VMPFC, and the peak of the categorical choice signal in motor cortex. Such differences would be expected if it were assumed that there is a temporal cost for translating signals in goods space into action space, and for conveying the results of computations from one brain region to another.

It is important to note that there are two differences between the comparison and sequential trials – both the imposition of a delay prior to the response, and the sequential vs. simultaneous presentation of options. These differences were selected as they captured some of the key differences between previous paradigms in which goods and action value signals had been observed in previous tasks. It is, of course, completely reasonable that investigators have designed paradigms more like our ‘comparison’ trial type [4], [14], [16]–[18], or like our ‘sequential’ trial type [4],[27],[28] – importantly, however, the signals they observe may lead them to different conclusions about the neural mechanisms of value-guided choice. Future work will be needed to refine precisely which of these two manipulations is most critical for pushing signals towards being found in one space or another. It is noteworthy, for instance, that in some experiments where options have been presented simultaneously but a delay is still imposed, goods-space value signals can still be isolated (albeit using different measures of neural activity) [16], [48].

In previous fMRI studies of sequential choice [10], VMPFC has been found to encode a goods value signal at the time of option presentation. At first sight, this appears discrepant with the absence of a goods value signal in VMPFC on our ‘sequential’ trials. Whilst a beta-gamma desynchronisation in VMPFC appeared to carry some information about the value of option 1, this did not reach statistical significance (figure S2). It is important, however, to consider the differences between what computational processes are likely to be visible to fMRI and MEG recordings. We have previously demonstrated that the MEG signal during goods value comparison can be modelled by the dynamics of competition in an excitation-inhibition network (EIN) [5]. This suggests valuation signals visible to MEG reflect trial-to-trial variability in this dynamic, competitive process. By contrast, the relationship between EIN activity and the BOLD fMRI signal is more complex, but it is related not only to local processing, but also to afferent input to a brain region [49]. One potential reconciliation of these findings is therefore that a goods value ‘afferent input’ signal is always present in VMPFC, and so can be seen in VMPFC fMRI signal, even when comparison can be found to take place in later, motoric structures [10]. By contrast, in situations when VMPFC supports comparison of options in goods space, this local processing is witnessed in both MEG dynamics [5] and also in fMRI value difference signals [16], [32], [50].

In summary, we have here presented evidence that when performing two formally identical decision tasks, the temporal evolution of value-related and choice signals differs depending upon how the information is revealed to subjects. If the value of each action is revealed separately, decision signals appear in an action-based frame of reference, reflected by beta desynchronisations in motor cortex. If both options are presented simultaneously, and subjects have to integrate across dimensions to form their decision, decision signals appear in an abstract frame of reference (chosen value minus unchosen value), reflected in beta synchronisations in VMPFC.

## Materials and Methods

### Experimental task

18 subjects (age range 21–33, 10 male, 8 female, recruited from the University of Oxford) repeatedly chose between two risky prospects, comprising differing reward magnitudes (represented by bar width) and probabilities (represented numerically), in order to obtain monetary reward (figure 1A). The probabilities of winning on each option were independent; thus, on any given trial, both, neither or either option(s) might yield reward. Stimuli were drawn such that reward magnitude and probability were never identical across the two options; subjects therefore needed to integrate across stimulus dimensions to make optimal choices. On some trials, however, both probability and magnitude were larger on one side than the other, a decision we classify as a ‘no brainer’.

On comparison trials, decisions were presented onscreen until a response was made. On sequential trials, one option was presented for 800–1200 ms jittered, followed by a 200–400 ms jittered delay, then the second option for 800–1200 ms jittered; subjects could respond only after removal of the second option. Stimuli were presented on either side of a fixation point; subjects selected the left option with a left-thumb button press, and the right option with a right-thumb buttonpress.

The difference between the two conditions was explained to the subjects in the instruction sheet thus: ‘For half of the decisions you have to make, you will see the screen as shown above (in figure 1A). In these trials, simply respond as soon as you feel that you have made your decision. For the other half of the decisions you have to make, you will see the two gambles one after the other, and then be presented with a screen displaying only a question mark. In these trials, you must wait for the question mark to appear before responding.’

On choosing a rewarded option, a ‘winnings bar’ displayed at the bottom of the screen increased in magnitude in proportion to the width of the chosen option. When this winnings bar reached a gold target on the far right of the screen, £2 was added to subjects' earnings, and the winnings bar reset itself to its original size. Total typical earnings for the task ranged from £26 to £34.

All subjects provided informed consent in accordance with local ethical guidelines.

### Behaviour: Fitting of subjective value functions

Subjective utility functions were derived from Prospect Theory [35], and were of the following form:
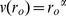


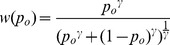
where *r_o_* and *p_o_* are the reward magnitude and probability of gaining reward, respectively, on outcome *o*. The subjective expected value of outcome o was calculated as:
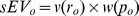
The probability of choosing each option was then calculated using a softmax choice rule:
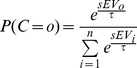
where *n* is the number of options (2 for this study) and *τ* is a temperature parameter that determines the stochasticity of action selection. Values of *α*, *γ*, and 1/*τ* (inverse temperature, denoted by *β* in results section) were fit by maximizing the likelihood of each subject's choices in the experiment, using non-linear fitting routines in MATLAB (The Mathworks, Natick, MA), separately for sequential and comparison trials. As in [5], we found that Bayesian Information Criteria (BIC) favoured Prospect theory over a simpler model that used objective probability and magnitude to compute expected value. A comparison between fitted parameter values in sequential and comparison trials is shown in figure 1C–E. The fitted values were used to calculate subjective expected values, which have been found to provide a better fit to neural data in value-guided decision tasks [51], [52], to use as trialwise regressors in analysis of MEG data.

### Behaviour: Logistic regression analysis

We used logistic regression to investigate the influence of *p_1_-p_2_* (probability difference between option 1 and 2), *r_1_-r_2_* (reward magnitude difference) and *EV_1_-EV_2_* (objective expected value difference) on the probability of choosing option 1 (see figure 1B). This was performed separately for each trial type. We normalised each explanatory variable before entry into the logistic regression (to ensure that parameter estimates were comparable across the different variables), and included a constant term to model any bias towards choosing one option over the other. For each explanatory variable and each trial type, we then performed a one-sample T-test across subjects' parameter estimates, to infer which variables had a significant effect on choice behaviour. We also performed a paired T-test between parameter estimates for sequential and comparison trials for each explanatory variable, to infer whether any variables had a greater or lesser influence on behavior between the two trial types.

### MEG/MRI data acquisition

MEG data were sampled at 1000 Hz on a 306-channel VectorView system (Elekta Neuromag, Helsinki, Finland), with one magnetometer and two orthogonal planar gradiometers at each of 102 locations distributed in a hemispherical helmet across the scalp, in a magnetically shielded room. A band-pass filter of 0.03–330 Hz was applied during acquisition. Head position was monitored at the beginning of each run, and at twenty-minute intervals during each run, using four head position indicator (HPI) coils attached to the scalp. Data were acquired in two or three runs, with pauses between blocks to save data acquired. HPI coil locations, headpoints from across the scalp, and 3 anatomical fiducial locations (nasion, left and right pre-auricular points) were digitized using a Polhemus Isotrak II prior to data acquisition. Simultaneous 60-channel electroencephalography data was acquired using a MEG-compatible EEG cap (ANT Neuro, Enschede, Netherlands), but is not discussed here. Vertical electrooculogram (EOG) and electrocardiogram were also measured to detect eye blinks and heartbeat, respectively. Stimuli were presented on a screen situated 1.5 meters away from the subject, inside the magnetically shielded room; stimuli were displayed via projector (refresh rate 60 Hz) situated outside the room. Stimulus presentation and timing was controlled using Presentation software (Neurobehavioral Systems, Albany, CA).

Magnetic resonance imaging (MRI) data for forward model generation were acquired using an magnetization-prepared rapid gradient echo (MP-RAGE) sequence on a Siemens 3T TRIO scanner, with voxel resolution 1×1×1 mm^3^ on a 176×192×192 grid, echo time = 4.53 ms, inversion time = 900 ms, recovery time = 2200 ms.

### MEG data pre-processing

External noise was removed from MEG data using the signal space separation method [53], and adjustments in head position across runs (detected using HPI) were compensated for using MaxMove software, both implemented in MaxFilter version 2.1 (Elekta Neuromag, Helsinki, Finland). Continuous data were down-sampled to 200 Hz and low-pass filtered at 40 Hz, before conversion to SPM8 format (http://www.fil.ion.ucl.ac.uk/spm). Eye blinks were detected from the EOG channel (EOG data was bandpass filtered at 1–15 Hz; local maxima lying more than 3 standard deviations from the mean were considered blinks). Detected eye blinks were used to generate an average eye blink timecourse, on which principle components analysis was run to obtain spatial topographies describing the average eye blink; these were regressed out of the continuous data (as per [54], without inclusion of brain source vectors as co-regressors; see http://www.fil.ion.ucl.ac.uk/~lhunt (‘Resources’ tab) for an SPM-based tutorial). Data were epoched with respect to stimulus onset (−1000 to 2000 ms around stimulus, with −200 to 0 ms pre-stimulus baseline), and button press (−2000 to 1000 ms around response, again with −200 to 0 ms pre-stimulus baseline). Artifactual epochs and bad channels were detected and rejected via visual inspection, using FieldTrip visual artifact rejection routines [55].

### MRI processing and forward modelling

All MRI processing and forward modelling was performed using SPM8. MRI images were segmented and spatially normalized to an MNI template brain in Talairach space; the inverse of this normalization was used to warp a cortical mesh derived from the MNI template to each subject's MRI space [56]. Digitized scalp locations were registered to head model meshes using an iterative closest point algorithm, to affine register sensor locations to model meshes [56]. Forward models were generated based on a single shell using superposition of basis functions which will approximately correspond to the plane tangential to the MEG sensor array [57]. The forward models are implemented in FieldTrip's *forwinv* toolbox [55].

### Beamformer source reconstruction

Source reconstruction was carried out using linearly constrained minimum variance (LCMV) beamforming [58] adapted for use on Elekta Neuromag data by using variance normalization between (magnetometer and planar gradiometer) sensor types, and dimensionality reduction to 64 spatial principal components [59]. This was used to reconstruct data to a grid across MNI space, sampled with a grid step of 7 mm. Full details of the beamforming approach used are given in [5]. The sensor covariance matrix was estimated separately for stimulus-locked and response-locked data using data pass band-filtered between 1 and 40 Hz, and 0% regularization.

### Whole-brain analysis of left minus right responses

In a preliminary whole-brain analysis, we looked for areas with greater beta power (13–30 Hz) on trials where the right button was pressed than on those where the left button was pressed, 500 ms–1000 ms after the last stimulus was presented (i.e. after option 2 was presented in sequential trials; after both options were presented in comparison trials). We performed this contrast at each of the beamformed voxels to produce a whole brain image, sampling the brain with a 7 mm gridstep. We then performed a one-sample T-test across subjects to produce the T-statistic images shown in figure 2A/3A (upsampled to 2 mm isotropic for display purposes). Inference was performed using a threshold of *p*<0.05 corrected voxelwise under assumptions of Gaussian Random Field theory.

We then beamformed data to the peaks from this analysis, and to a VMPFC peak identified in a previous paper [5], to perform time-frequency regression in order to test for correlates of value in these areas.

### Time-frequency regression of source-reconstructed data

We used multiple regression to estimate the contribution of the value of each option and the response made to power in each frequency band at each timepoint through the decision. In the sequential trials, at the time of option 1 presentation (figure 2B/E), we included the value of this option as the regressor (and searched in contralateral M1 for responses). The full regression model at each timepoint and frequency band therefore consisted of three terms – a constant (*β_0_* in regression model below), the effect of the value of option 1 (*β_1_* below), and a categorical term reflecting which option was chosen (*β_2_*). At the time of option 2 presentation (figure 2C/F), we included the (action-space) value difference between contralateral and ipsilateral options (and calculated the differential response in contralateral minus ipsilateral M1). The full regression model consisted of four terms – a constant (*β_0_*), the value of contralateral (*β_1_*) and ipsilateral (*β_2_*) options, and a categorical term reflecting which option was chosen (*β_3_*). The effect of action value difference was estimated by performing a contrast of parameter estimates for *β_1_ and β_2_*. In the comparison trials, we performed the same action-space analysis in M1 (figure 4B); and a goods-space analysis in VMPFC, in which we included the value difference between chosen and unchosen trials (figure 6A/B/D/E), separately for harder and nobrainer trials. Again the full regression model consisted of four terms – a constant (*β_0_*), the value of chosen (*β_1_*) and unchosen (*β_2_*) options, and a categorical term reflecting which option was chosen (*β_3_*). The effect of goods-space value difference was estimated by performing a contrast of parameter estimates for *β_1_ and β_2_*. We also performed the same analysis for harder sequential trials (figure 6C/F). Importantly, in all regressions, the inclusion of the final decision regressor as a covariate allowed us to isolate the variance that could be explained by value independent of choice. Value regressors were normalized prior to regression, so they occupied a similar range of values across subjects.

At each trial, the source-reconstructed data **d(r_i_)** was decomposed into 40 time-frequency bins linearly spaced between 1 and 40 Hz, by convolving the data with Morlet wavelets (Morlet factor 5) [60]. This yielded, at each trial *tr*, frequency *f*, and timepoint *t*, an instantaneous estimate of the power at that frequency. Linear regression was then used to estimate the contribution of the *n* explanatory variables (EV) to this estimated power:
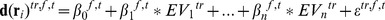
where ε is the residual from the regression. The parameter estimates *β_1…n_*, normalized by their variances, were submitted to a group-level one-sample T-test to test for significant effects of each explanatory variable.

For statistical inference on the effects of overall value and value difference on region of interest data, we performed a cluster-based permutation test at the group level. The logic of this permuation test is identical to that used in non-parametric statistical inference of cluster sizes in functional MRI and other MRI based analyses [61]. We generated 5000 randomly permuted T-statistics for each timepoint and frequency bin, by randomly sign-flipping the group design matrix 5000 times. We then thresholded each permutation's time-frequency decomposed T-statistic map at a threshold of T(17)>2.0, and measured the maximum size of any cluster passing this threshold in the map, to build a null distribution of cluster sizes. We then compared the size of clusters from the true T-statistic map to those from the null distribution. We report clusters at a significance level of *p*<0.05, corrected for multiple comparisons across time and frequency.

## Supporting Information

Figure S1 In ‘sequential’ trials, the value of option 1 is encoded earlier than the value of option 2 in motor cortex beta desynchronisation, at the time of option 2 presentation. The regression coefficient of VOpt1 is shown in blue (arbitrary units, mean +/− 95% confidence intervals across subjects); the effect of VOpt2 is shown in green.
(EPS)

Figure S2 VMPFC shows no significant value coding in action space, or coding of action. *Layout is equivalent to main* *figure 5*. (A) Correlates of the value of option 1 at time of option 1 presentation. Color represents T-statistic. The beta-gamma desychronisation at approximately 700 ms–1100 ms does not quite survive cluster correction. (B) Correlates of the action value difference at the time of option 2 presentation. (C) Contrast of trials on which the chosen option is contralateral vs. ipsilateral, at the time of option 2 presentation. (D) Timecourse of beta band correlates of value of contralateral option (blue and choice (red) at time of option 1 presentation. Lines represent mean +/− 95% confidence intervals across subjects. (E) Timecourse of beta band correlates of action value difference (blue) and choice (red) at time of option 2 presentation. (F) Correlates of action value difference in comparison trials, timelocked to the response. (G) Contrast of trials on which chosen option was contralateral vs. ipsilateral in comparison trials, timelocked to thre response.
(EPS)

Figure S3 pSPL shows no significant value coding in action space, or coding of action. *Layout is equivalent to main* *figure 5*. (A) Correlates of the value of option 1 at time of option 1 presentation. Color represents T-statistic. (B) Correlates of the action value difference at the time of option 2 presentation. (C) Contrast of trials on which the chosen option is contralateral vs. ipsilateral, at the time of option 2 presentation. (D) Timecourse of beta band correlates of value of contralateral option (blue and choice (red) at time of option 1 presentation. Lines represent mean +/− 95% confidence intervals across subjects. (E) Timecourse of beta band correlates of action value difference (blue) and choice (red) at time of option 2 presentation. (F) Correlates of action value difference in comparison trials, timelocked to the response. (G) Contrast of trials on which chosen option was contralateral vs. ipsilateral in comparison trials, timelocked to the response.
(EPS)
